# Development and validation of an ^18^F-FDG PET/CT radiomic nomogram for predicting axillary lymph-node status after neoadjuvant chemotherapy for breast cancer: a multicenter study

**DOI:** 10.1007/s12149-025-02099-4

**Published:** 2025-08-17

**Authors:** Yu Li, Kun Chen, Luqiang Jin, Hailin Huang

**Affiliations:** 1 Department of Nuclear Medicine, Cancer Center, Zhejiang Provincial People’s Hospital (Affiliated People’s Hospital), Hangzhou Medical College, 158 Shangtang Road, Hangzhou, Zhejiang China; 2 Zhejiang Cancer Hospital, Hangzhou Institute of Medicine (HIM), Chinese Academy of Sciences, Hangzhou, 310022 Zhejiang China

**Keywords:** ^18^F-FDG PET/CT, Radiomics, Breast cancer, Axillary lymph nodes, Neoadjuvant chemotherapy

## Abstract

**Rationale and objective:**

To develop and validate the predictive value of ^18^F-FDG PET/CT radiomics models based on data preprocessing methods for axillary lymph-node (ALN) status after neoadjuvant chemotherapy (NAC) for breast cancer.

**Materials and methods:**

According to the status of ALN after NAC, we divided the breast cancer patients of the three scanners into the pathological complete remission (pCR) and non-pCR groups, respectively. Totally 630 models were obtained based on various data preprocessing, feature filtering, and modeling approaches. On the one hand, different data preprocessing methods were compared to analyze the advantages of different preprocessing methods. On the other hand, the AUC of predicting ALN status was compared among all models, and the model with the best prediction was obtained. Finally, the optimal model is combined with the clinical and the corresponding Nomogram is plotted.

**Results:**

The comparison of the data preprocessing modalities revealed that the model prediction of tumor-to-liver ratio (TLR) radiomics was better than origin radiomics (OR), and the effect of Combat and Limma was better than without batch effects. All preprocessing modalities could be used as a potential method that can further optimize the model. The optimal model had a predicted AUC of 0.798 for ALN status after NAC for breast cancer in the test set and an AUC of 0.811 when combined with clinical characteristics.

**Conclusion:**

It is necessary to pre-process the data before conducting a study on multicenter data, and the model developed in this way can effectively predict the status of ALN after NAC in breast cancer.

**Supplementary Information:**

The online version contains supplementary material available at 10.1007/s12149-025-02099-4.

## Introduction

Breast cancer has been one of the malignant diseases that threaten women’s health and lives. NAC is a conventional treatment modality for breast cancer, intended to control the lesions and metastases through different cycles of preoperative chemotherapy, to reduce the clinical stage, and to create conditions for a successful surgery [1–3]. Currently, most breast surgeries still tend to pursue R0 resection (complete removal of the tumor with the negative microscopic observation of the incision margin, i.e., no tumor residue), and axillary lymph-node dissection (ALND) is also used to better achieve R0 resection [4, 5]. However, as we know, ALND can cause a variety of complications, the most common of which is breast cancer-associated lymph-node edema [6–8]. Many studies have shown that the substitution of ALND with sentinel lymph-node biopsy (SLNB) has no impact on patient survival if there is no metastasis, while SLNB has fewer complications and is helpful in improving the quality of patient survival after surgery [9, 10]. Therefore, if the status of ALN pCR after chemotherapy can be predicted before NAC, it is instrumental in the development of the treatment regimen, the selection of surgical options, and the improvement of the patient’s quality of life after treatment.

Radiomics is the high-throughput extraction of a large amount of image information from images (CT, MRI, PET, etc.) to achieve tumor segmentation, feature extraction, and model building, and to assist physicians in making the most accurate diagnosis by virtue of deeper mining, prediction, and analysis of image data information [11–14]. ^18^F-FDG PET/CT is a sensitive hybrid imaging method for staging breast cancer patients and for assessing treatment response[15, 16]. Currently, some studies have attempted to predict ALN pCR status after NAC for breast cancer using MRI and ultrasound radiomics with meaningful results but not yet using ^18^F-PET/CT radiomics [17–19]. Meanwhile, NCCN guidelines have increased the applicability of PET/CT imaging in breast cancer, and more patients will undergo preoperative PET/CT imaging, which makes breast cancer PET/CT radiomics studies more valuable and possible for clinical utilization [20].

Research on the expansion of radiomics methods has focused on the selection of volumes of interest and segmentation methods, feature selection, etc. The volumes of interest currently mainly include primary tumors, metastases, and paracancerous areas [21–24]. The segmentation methods mainly include manual segmentation and threshold segmentation. The feature selection mainly includes the selection of appropriate subsets from the feature set directly or after combination transformation according to different evaluation criteria. All of these studies have contributed to the improvement of model performance, but there is a paucity of more studies and data harmonization methods that can multi center the application of model results, and this has hindered the clinicalization of radiomics to some extent. Considering the high sensitivity of radiomics, differences between patients and scanners in different centers are the main problems affecting multicenter applicability, so it is necessary to harmonize these differences or propose a data preprocessing method.

There are numerous studies that demonstrated TLR is better than SUVmax at assessing treatment response; many ^18^F-PET/CT-related breast cancer studies also use TLR as an indicator at present [25–27]. The batch effect is an experimental error caused by different times, operators, and instruments, independent of the biological or scientific variables in the study, and has a significant effect on high-throughput sequencing data [28, 29]. In the present study, we attempted to eliminate interindividual and batch variation through TLR radiological characterization and batch harmonization methods, and enhance the robustness of the model through discretization, to explore the role of pre-treatment pet/ct radiomics in predicting ALN status after NAC for breast cancer while validating the role of these data preprocessing modalities in multicentre data while, and in consequence to advance the clinicalization of radiomics.

## Materials and methods

### Patients

No residual tumor in the final pathology for ALN after NAC was considered pCR of ALN. A total of 147 breast cancer patients from the Zhejiang Cancer Hospital (29 patients) and the Zhejiang Provincial People’s Hospital (118 patients) were included in this retrospective study, all of whom underwent ^18^F-FDG PET/CT imaging before NAC. The inclusion criteria were as follows: (1) First diagnosis of breast cancer and having a baseline ^18^F-FDG PET/CT imaging. (2) ^18^F-FDG PET/CT imaging demonstrated the presence of ALN metastasis. (3) Preoperatively treated with NAC. (4) Postoperative pathological results of the primary tumor and all ALN after ALN dissection were available. The exclusion criteria were: (1) the breast has been surgical in the past; (2) combination of other cancers; (3) bilateral breast cancer, as shown in Supplementary Fig. A. All NAC regimens were developed and implemented following the NCCN guidelines and all procedures in studies involving human participants were conducted by the 1964 Helsinki Declaration and its later amendments or comparable ethical standards. The Ethics Committee of the hospital approved this study.

### Image acquisition

PET/CT images were derived from three scanners. The images of scanners 1 and 2 were, respectively, obtained from the uMI Panorama (Shanghai, China) and the Siemens Biograph 64 PET/CT (Munich, Germany). The images of scanner 3 were acquired with a GE Discovery Elite scanner (Chicago, America). Patients were instructed to fast for 6 h and to control fasting blood glucose below 10 mmol/L prior to intravenous administration of the imaging agent. ^18^F-FDG imaging agent was injected at a dose of 0.1–0.12 mCi per kilogram of body weight, followed by 1 h of patient rest in a quiet environment. During this time, patients were permitted to dink 800–1000 ml of water to facilitate stomach dilation and bladder emptying. All patients were scanned from the base of the skull to the distal femur. The GE spiral CT scan was performed with automatic mAs, a tube voltage of 120 kV, and a layer thickness of 5 mm. The PET was scanned in seven-bed positions, and each bed position need 1.5 min. The ordered-subset expectation–maximization (OSEM) iterative algorithm was used to reconstruct the PET images with CT values being used for attenuation correction. The Siemens CT images was obtained using a spiral CT scan with a tube voltage of 120 kV, automatic mAs and layer thickness of 5 mm. The PET was scanned in seven-bed positions and each bed position need 1.5 min. The ordered-subset expectation–maximization method was used to iteratively recreate the PET scans in three dimensions while attenuation correcting the PET images using information from CT scans. The spiral CT of uMI Panorama scan was acquired with automatic mAs, a tube voltage of 120 kV, and a layer thickness of 5 mm. The PET was scanned in seven-bed positions, and each bed position need 1.5 min. The OSEM method was used to recreate the PET images with attenuation correction from CT scans. All data were independently analyzed by two nuclear medicine physicians with over 5 years of experience in PET/CT diagnosis. The region of interest was delineated based on the lesion’s contour. The two physicians reviewed the images, engaged in discussions, and ultimately reached a consensus to determine the measurement results.

### Image feature extraction

First, all images were imported into the 3D slicer in DICOM format, and the CT and PET images of the primary tumor and the PET images of the normal liver were segmented. The segmentation of the CT images of the primary tumor was performed by the layer-by-layer manual outline method, the PET images were performed by the SUVmax 40% threshold method, and the PET images of the liver were selected as the volume of interest for 20 cm^3^ located away from the hepatic hilum. As shown in Supplementary Fig. B. To reduce interindividual variability and to exclude unstable radiomic features, two nuclear medicine doctors segmented the volume of interest without knowledge of pathology, with one of them segmenting it twice at different times. Then, the voxel resampled images of 1 cm^3^ were extracted radiomics features by 3 d Slicer’s SlicerRadiomics extensions. SlicerRadiomics provides a 3D Slicer interface to the pyradiomics, pyradiomics is an open-source python package for the extraction of radiomics features from medical imaging and is IBSI accredited. Laplacian of Gaussian (LoG) and wavelet filtering were used to pre-process PET and CT images, where wavelet filtering consisted of a combination of high-pass (H) and low-pass (L) filtering in each dimension based on PET/CT images, including Wavelet-LLH, Wavelet-LHL, Wavelet-LHH, Wavelet-HLL, Wavelet-HLH, Wavelet-HHL, Wavelet-HHH, and Wavelet-LLL. Then, First-order, Gray-Level Co-occurrence Matrix (GLCM), Gray-Level Dependence Matrix (GLDM), Gray-Level Run-Length Matrix (GLRLM), Gray-Level Size Zone Matrix (GLSZM), and Neighboring Gray Tone Difference Matrix (NGTDM) features were extracted from the original and pre-processed images. Finally, the CT and PET radiomic features of the primary tumor were combined into OR. The PET radiomic features of the primary tumor in addition to the volumetric features were divided by the PET radiomic features of the liver and combined with the CT features of the primary tumor to form the TLR radiomic features. The three sets of data obtained from two nuclear medicine doctors were subjected to an ICC test between every two groups and were considered to have good reproducibility and reliability when ICC > 0.75.

### Batch effects and intensity discretization

Batch effects are sub-groups of measurements that have qualitatively different behavior across conditions and are unrelated to the biological or scientific variables in a study. Both Limma and Combat are batch adjustment methods for biological information analysis and genomics data development. Combat is currently used in multicenter radiomics studies, but Limma is not widely used. With Combat and Limma harmonization, we attempted to reduce the variability of external factors, such as the effects of different scanners, to verify whether these harmonization methods would contribute to enhancing the effectiveness of the multicenter model.

Considering the high sensitivity of radiomics, we need to improve the stability of the model and enhance the robustness of abnormal data. With reference to the approach of discretizing the images before extracting parameters, we discretized the data after feature extraction. Radiomics features were discretized using fixed bin number (FBN, using 32 and 64 bins) and fixed bin width (FBW, using 32 and 64 bins). The FBN is discretized according to the magnitude of the values of the feature parameters, with an equal number of bins each. For feature *A*, *A*(*x*) represents the feature parameters of patient *x*, min(*A*) represents the smallest parameters in feature *A*, Rank(*A*(*x*)) represents the ranking of *A*(*x*) after ascending ranking, and Counta(*A*) represents the number of features A parameters, n is the number of discrete bins, and y is the new value of A(*x*) after discretization, as shown in the following formula:

If $$A(x) = \min (A)$$

$${\text{y}} = 1$$

If $$A(x) \ne \min (A)$$

$$y = \left\lceil {n \times \frac{{{\text{Rank}}(A(x)) - 1}}{Counta(A) - 1}} \right\rceil$$

The FBW is discretized according to the difference between the maximum and minimum values of the feature parameters, with an equal width per bin. In addition to those mentioned above, the max(*A*) represents the largest parameters in feature *A*, as shown in the following formula:

If $${\text{A}}(x) = m{\text{in}}(A)$$

$${\text{y}} = 1$$

If $$A(x) \ne \min (A)$$

$$y = \left\lceil {n \times \frac{A(x) - \min (A)}{{\max (A) - \min (A)}}} \right\rceil$$

### Nomogram

First, we multiplied each valid feature with the weights and summed all the results to obtain the radiomics score. Then a multi-factor regression model was constructed, which included radiomic scores and meaningful ER, PR, and HER2 in the clinical data. According to the degree of contribution of each influential factor in the model to the outcome variable (the magnitude of the regression coefficient), a score was assigned to each value level of each influential factor, and then, the individual scores were summed to obtain the total score, and the predicted value of the outcome event for that individual was calculated by the functional transformation relationship between the total score and the probability of the occurrence of the outcome event. Finally, the complex regression equation is transformed into a visual graph to make the results of the prediction model more readable and to facilitate the assessment of patients.

### Statistical analysis

**Fig. 1:**
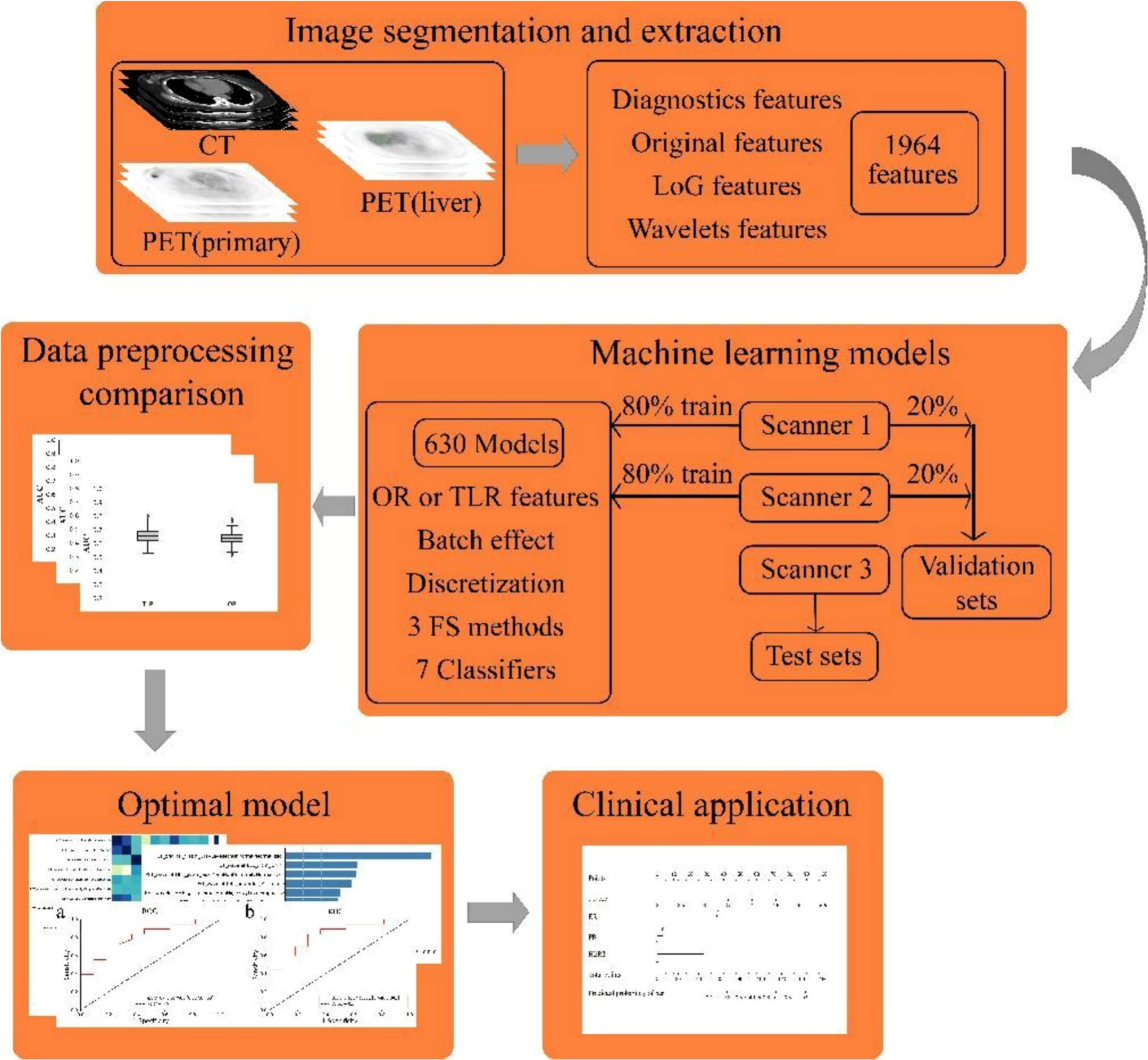
The workflow of data processing and machine learning

We subjected features to the Pearson test between every two and removed features with a correlation > 0.9. Then, we predicted 630 model effects based on (1) whether the feature type was TLR radiomics or OR, (2) the batch effect processing method, without processing, Limma or Combat, and (3) the discretization method, without discretization, FBW32, FBW64, FBN32, FBN64, and (4) differences in selection of valid features and modeling methods, as shown in Fig. 1 workflow. Moreover, to reduce the effect of chance and improve the reliability and stability of the method, we repeated the prediction process for all models 1000 times and averaged the results as follows [30]: We divided the patients of scanners 1 and 2 into non-pCR and pCR groups according to the status of ALN after NAC, and then down-sampled the non-pCR group according to the number of pCR group (95:52), and randomly selected 80% of the two down-sampling groups as the training set and the remaining 20% as the validation set, and included the patients of scanner 3 as the test set. The features are filtered by Decision Tree (DT), Random Forest (RF), and XGBoost, and the top ten most important features are retained as valid features according to the importance score. Finally, we attempted to construct the prediction models by seven classifiers, DT, Extra Trees (ET), K-Nearest Neighbor (KNN), RF, Support Vector Machine (SVM), XGBoost, and Stochastic Gradient Descent (SGD) and calculated the AUCs of the validation and test sets based on the model prediction results, the average value of the AUC of the two sets is taken as the final prediction effect of the model.

Based on the prediction results of all models, we (1) compare multiple data preprocessing methods and (2) select the model with the best prediction results. To enhance the credibility of the small data size sample model, we performed permutation tests for the highest and lowest accuracy of the best-performing model. The significance of the clinical characteristics of the three scanners between the pCR and non-pCR groups was verified by the Chi-square test and t test. The optimal model was combined with meaningful clinical features to validate the predictive effect of the radiomics + clinical features model, and the results were presented as Nomograms to facilitate clinical application. The processing of batch effects and the generation of Nomograms were all performed by R studio. The rest of the radiomics processing and image generation and comparison between groups was implemented in Python.

## Results

### Clinical characteristic

The characteristics of the patients who participated in the study are shown in Supplementary Table A. In this study, we included 147 patients with a pathological diagnosis of breast cancer with ALN metastasis and who had undergone preoperative NAC. Depending on the difference in scanner devices, we divided the patients into three groups. Then, according to the different statuses of ALN after NAC, we divided the patients of each scanner into the pCR group and the non-pCR group. Overall, ER and molecular subtypes in scanners 1 and 2, and PR and HER2 in scanner 2, were meaningful for the identification of the pCR and non-pCR groups.

### Comparison of preprocessing methods

**Fig. 2:**
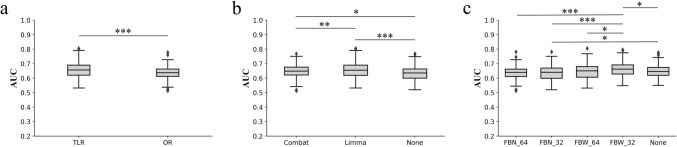
A comparison of different data preprocessing methods. **a** Comparison of OR and TLR radiomics. **b** A comparison of the Limma and Combat harmonization methods, with none as not eliminating the batch effect. **c** Comparison between different discretization methods; none means not discretized. *p < 0.05, **p < 0.01, ***p < 0.001

We extracted 982 features each from the VOI of CT images and PET images, respectively, and combined them to obtain a total of 1964 features. After removing the features with ICC < 0.75, 1266 features were retained. Among the 630 models, 320 each used OR and TLR radiomics, 210 each used Combat and Limma to process batch effects as well as no batch effects, and 126 each used FBN (32, 64) and FBW (32, 64), as well as no discretization, and the corresponding box plots, were plotted according to the AUC of the predicted effects of the models, as shown in Fig. 2. The mean AUC of the TLR radiomics model was 0.654 (95%CI 0.559 ~ 0.758), which was better than the mean AUC of the OR model of 0.635 (95%CI 0.544 ~ 0.721), with a significant difference (*p* < 0.001). The mean AUC of 0.655 (95%CI 0.563 ~ 0.777) for the model after Limma harmonization was significantly different from both the mean AUC of 0.646 (95%CI 0.554 ~ 0.731) for the model after Combat harmonization and the mean AUC of 0.633 (95%CI 0.539 ~ 0.722) for the model without batch effect processing (*p* = 0.007, *p* < 0.001). Likewise, the mean AUC of the model harmonized by Combat was significantly different from the mean AUC of the model without processing batch effects (*p* = 0.044). In the comparison of discretization methods, the mean values of AUC for the five approaches of FBN64, FBN32, FBW64, FBW32, and no discretization were 0.637 (95%CI 0.546 ~ 0.721), 0.635 (95%CI 0.539 ~ 0.719), 0.644 (95%CI 0.554 ~ 0.748), 0.660 (95%CI 0.564 ~ 0.761), and 0.648 (95%CI 0.573 ~ 0.740), respectively. Among them, FBW32 was the most effective and significantly different from the other four methods of processing (*p* < 0.05), and there was also a significant difference between FBN32 and no discretization (*p* = 0.033).

### Comparison and analysis of models

**Fig. 3:**
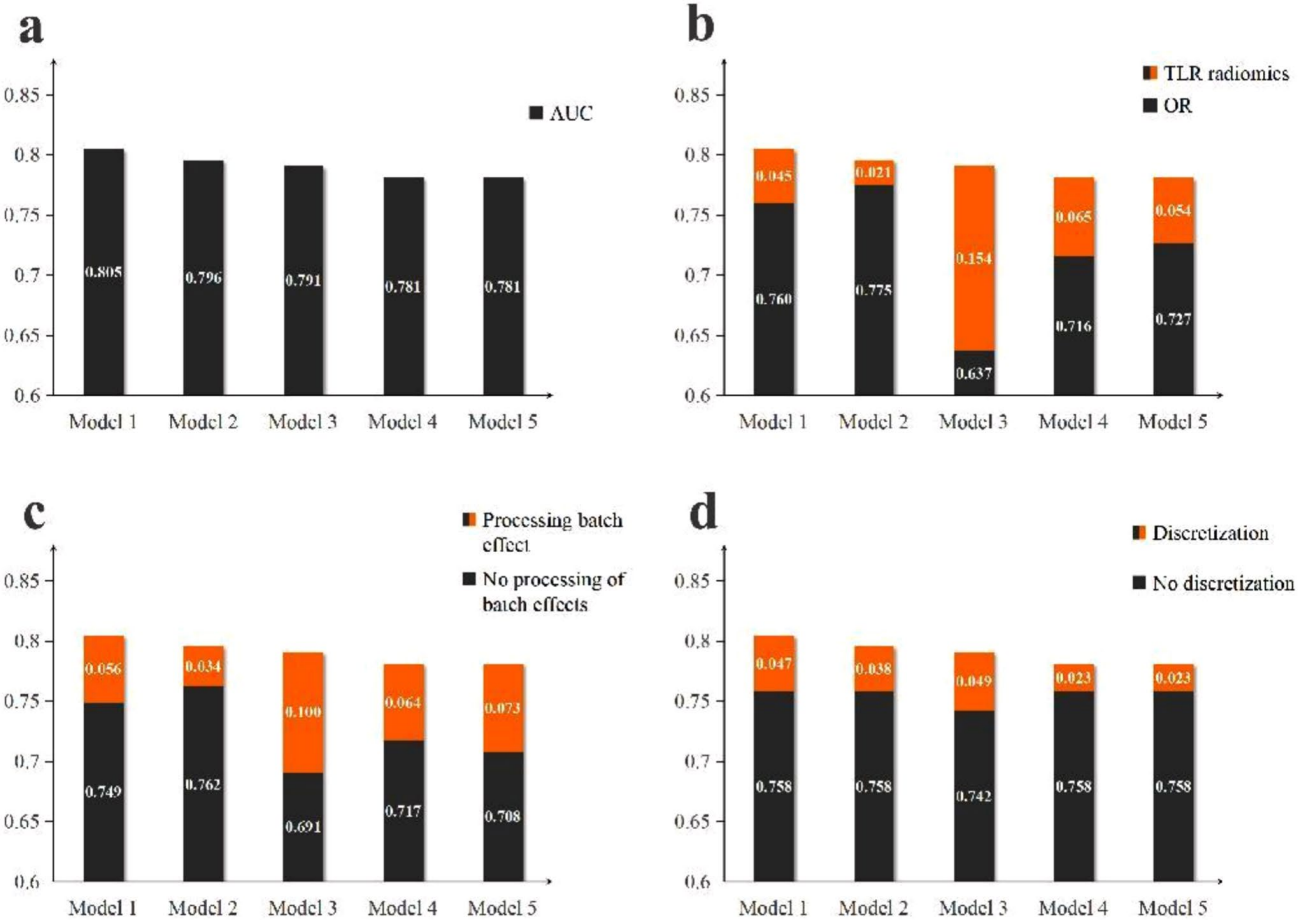
The effect of different data preprocessing methods on the five models with the best prediction results. **a** AUC of the five models with the best predictions. **b** Effect of replacing OR with TLR radiomics on the five models with other preprocessing modalities is unchanged (the orange part is the increase in AUC after replacing with TLR radiomics). **c** Effects on the five models after processing the batch effect (the orange part represents the increased AUC after processing the batch effect). **d** The effect of discretization on the five models (the orange part represents the increased AUC after discretization)

Referring to the average of the AUC of each model validation set and test set, we selected the five models with the best prediction results, and their specific data preprocessing and model construction are shown in Table 1. The mean value of AUC predicted by model 1 in the validation and test sets was 0.805, which was the most effective among all models, and 0.796, 0.791, 0.781, and 0.781 for Model 2–5, respectively. We performed DeLong’s test to compare the AUC of Model 1 with those of Models 2–5, yielding *z* values of 0.306, − 0.419, − 1.995, and − 2.197, with corresponding *p* values of 0.621, 0.337, 0.023, and 0.014, respectively. The results indicated no statistically significant difference between Model 1 and Models 2–3. However, since Model 1 achieved the highest AUC, it was selected for all subsequent analyses. With all other procedures consistent, replacing OR with TLR radiomics improved the prediction of these five models by a range of 0.021–0.154. Similarly, processing batch effects (0.034–0.100) and discretization (0.023–0.049) could improve the model predictions to different extents, as shown in Fig. 3.

**Table 1:** Top 5 models in AUC ranking

	Feature type	Batch effect	Discretization	Feature selection	Model
Model 1	TLR	Limma	FBW (64bins)	RF	SVM
Model 2	TLR	Limma	FBW (32bins)	RF	SVM
Model 3	TLR	Limma	FBW (32bins)	DT	SVM
Model 4	TLR	Limma	FBN (32bins)	RF	SVM
Model 5	TLR	Limma	FBN (64bins)	RF	SVM

### Optimal model

Before screening all models for valid features, correlation tests were performed between every two features (retaining features with correlation coefficients < 0.9), and ultimately, 493 features of model 1 were retained. According to the weight ranking of the valid features after RF screening, we reserved the top 10 features with the highest weights as the valid features of Model 1. The feature names and weights are shown in Supplementary Fig. C, and the correlations heat map among the features are shown in Supplementary Fig. D. Model 1 predicted an AUC of 0.812 and 0.798 in the validation and test sets, respectively. To further ensure the credibility of the small sample size model, we did permutation tests in the conditions of minimum and maximum precision of the model. The Sobs value of the permutation test for the minimum accuracy is 0.340 (*p* < 0.01) and the Sobs value for the maximum accuracy is 0.520 (*p* < 0.001), and the results are shown in Supplementary Fig. E.

**Fig. 4:**
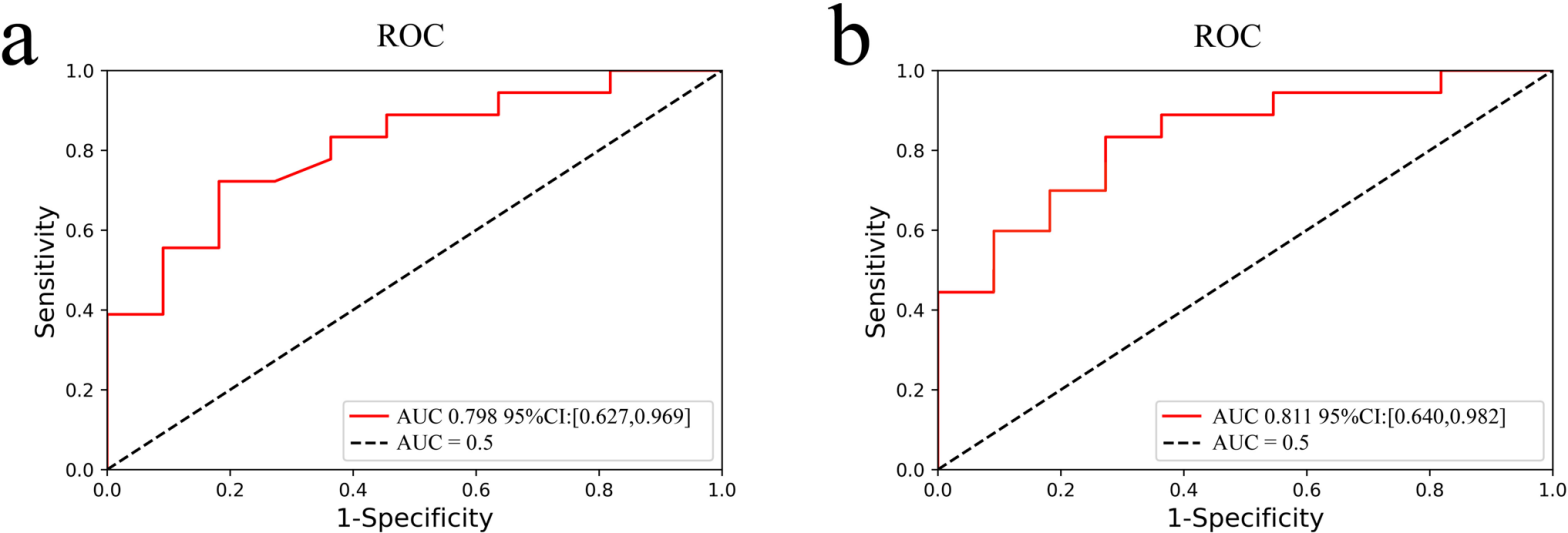
ROC curves predicted by Model 1 and radiomics + clinical characteristics model in the test set. **a** ROC curve of Model 1. **b** ROC curve of Radiomics + Clinical Characteristics model

Then, we combined the meaningful ER, PR, and HER2 of clinical characteristics with the valid radiomic features in Model 1, and validated the predictive effect of this radiomics + clinical model in an SVM classifier consistent with Model 1. The AUC predicted by the radiomics + clinical model in the test set was 0.811, and the ROC curves predicted by model 1 and the radiomics + clinical model in the test set are shown in Fig. 4.

Finally, according to the feature specific values and the weight of each feature during RF screening, we calculate the radiomics score. F_1_-_10_ represents the different feature specific values, W_1-10_ represents the weights of the features, and r-score is the radiomics score with the following formula:

$$r - score = F_{1} \times W_{1} + F_{2} \times W_{2} + \cdots F_{10} \times W_{10}$$

**Fig. 5:**
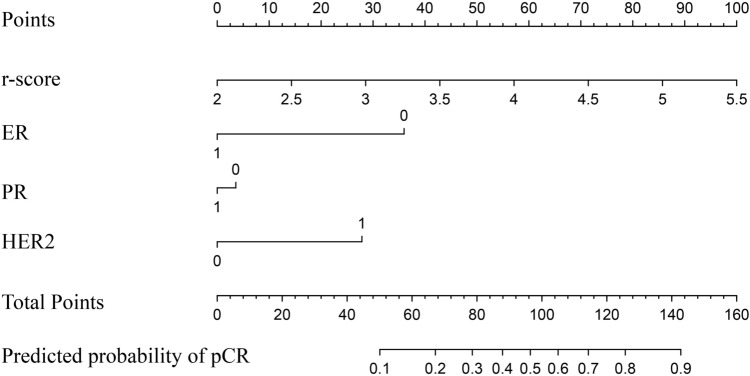
Radiomics + clinical characteristics model of Nomogram

The radiomics + clinical model was simplified to a combination of radiomic scores and meaningful clinical characteristics, and the results were presented in the form of a Nomogram, as shown in Fig. 5.

## Discussion

Preoperative NAC is one of the main treatment modalities for breast cancer patients, which can downsize the lesion or reduce the clinical stage of the patient to reduce the scope of surgery and improve the prognosis of the patient [31]. In addition to the size of the primary tumor, the status of the ALN is also a key concern after NAC, which can influence the clinical staging and thus further influence the ALN surgery approach [32]. This study attempted to predict the status of ALN in breast cancer patients after NAC using ^18^F-FDG PET/CT imaging before NAC. There are many studies predicting ALN status using post-NAC imaging or both pre- and post-existing imaging, but a few studies use only pre-NAC imaging. Prediction with PET/CT imaging before NAC not only predicts preoperative ALN metastasis but also informs the choice of the treatment regimen, especially when the prediction is informed that NAC is not effective against the primary tumor.

Another focus of this study is to verify whether multiple data preprocessing approaches contribute to the multicenter generalization of the model. As we know, radiomics uses high-throughput data, resulting in a high sensitivity that helps to preserve features that are not obvious to VOI but are equally susceptible to differences caused by objective factors (e.g., scanner model, etc.). SUV is influenced by factors, such as blood glucose level, the physical condition of the subject, post-injection development time, and clearance of ^18^F-FDG in the blood circulation [33–35]. To reduce the influence of these factors, some scholars have introduced the concept of TLR into the diagnosis, efficacy assessment, and prognosis of tumors, and even considered this index to be more accurate than SUVmax. Park et al. concluded that among many PET parameters, SUVmax normalized to liver uptake after neoadjuvant chemoradiotherapy was the best predictor of the pathologic complete response [36]. Song et al. concluded that TLR can provide useful information in treatment prognosis for hepatocellular carcinoma patients treated with locoregional therapy [37]. In this study, we attempted to apply TLR to radiomics by extracting parameters from PET images of the tumor and 20 cm^3^ normal liver tissue separately, and then dividing tumor PET features (except volume features) by PET features of normal liver tissue to obtain TLR radiomics features. The aim was to further reduce the effects of factors such as blood glucose level and clearance of ^18^F-FDG in the blood circulation between different patients by this data preprocessing. The overall prediction of the 320 TLR radiomics models in this study was superior to that of the OR model, and TLR radiomics also improved the prediction of the 5 superior models with different degrees of improvement (0.021–0.154). This result is similar to the prediction of disease-free survival in cervical cancer by Ferreira et al. in a multicenter [38].

The differences in scanner types, parameter settings, and reconstruction methods among different centers are also important factors in the prediction effectiveness of the model. To enhance the model’s generalizability across multiple centers, it is crucial to minimize the impact of image heterogeneity as much as possible. This study attempted to reduce the batch effect by two data preprocessing methods, Combat and Limma, and to verify the effect of these two methods on model optimization. Combat is a classical Bayesian-based analysis that applies known batch information for the batch correction of high-throughput data and is suitable for small sample size studies [39–41]. Although ComBat is mainly used for Bioinformatics analysis, its applicability conditions fit well with the characteristics of radiomics data, and so it is also used in radiomics to reduce batch effects in multicenter data. The prediction of all models processed with Combat in this study was better than the models without batch effect processing, further confirming the applicability of the method in radiomics. Limma is also one of the methods mainly used for Bioinformatics analysis to eliminate batch effects. We have attempted to apply this method to radiomics and demonstrated that it is superior to Combat, but whether Limma is better only for this study or for multiple disease studies remains to be demonstrated in more studies. Overall, reducing batch effects is an essential data preprocessing approach for multicenter data in radiomics studies, whether it is Combat or Limma, which also helps mitigate the impact of inter-scanner image heterogeneity on model generalizability.

It is recognized by many scholars due to the features such as increased computing speed by discretization, strong robustness of features to anomalous data, and stronger stability of the model. In general, radiomics studies are discretized based on Hu of CT images and SUV of PET images, which inevitably lose some of the image information [42]. Not only that, we would resample voxels to a size of 1 cm^3^ before extracting parameters from CT and PET images, and if the resampled PET images are discretized, then features are extracted, and the amount of calculation will be large, so many studies with PET/CT images are only discretized and further studied on PET images [38, 43]. Therefore, we attempted to discretize the parameters after extracting features with equal width and frequency, which could preserve more image features and provide the model with the advantage of discretization, as well as study both CT and PET images. The results show that different discretization methods have different effects, and it is not possible to conclude the advantages and disadvantages of different discretization methods from this study alone, and more studies are needed for further validation. The possible reason is that FBN is more adaptable to uneven data distribution (such as long-tail distribution) and can retain more detailed information, while FBW is more stable for uniformly distributed data, such as in the case of a uniform threshold. FBW divides features based on a uniform physical scale (such as the Hu value in CT or the SUV value range in PET), which may be more suitable for the contrast pattern between lesions and background in this study. However, it is certain that discretization is helpful for further optimization of the model.

There are still some limitations in this study. To further the clinicalization of radiomics, every process of our study has been attempted to be as objective and standardized as possible. However, due to technical limitations, there is a lack of a method to obtain VOI with full objectivity and accuracy currently, and it is still necessary to obtain VOI of lesions semi-automatically or even manually, which will hinder the clinicalization of radiomics to some extent. Moreover, although data preprocessing was performed in terms of multicenter data harmonization and the model effect was relatively satisfactory, prospective experiments in more centers are needed to truly guide clinical practice. Furthermore, as we know, the preferred NAC regimens and efficacy are different for different breast cancer subtypes (according to ER, PR, and HER2 status), and there is a lack of sufficient patients to support a more detailed study of the same subtype and the same treatment regimen, which will be further refined in the future when more patients are available or data from more centers are included.

In spite of some limitations, the innovative and clinical value of the study is undeniable. First, we are the first to predict the status of ALN after NAC for breast cancer with PET/CT radiomics, which fills the gap of ^18^F-FDG PET/CT research in this direction. Second, although a small number of studies have used TLR radiomic features, this is the first time that they have been used to predict ALN pCR after NAC for breast cancer. Finally, three types of data preprocessing were performed for interindividual, inter-group (different scanners), and individual outliers to make the radiomic features more representative of the pathological characteristics of the primary tumor. This provides a new idea of data preprocessing for future radiomics studies.

## Conclusion

In this study, we performed a total of 630 ^18^F-FDG PET/CT-based radiomics models to predict ALN pCR after NAC for breast cancer. We selected an optimal model by comparing the differences between different data preprocessing methods (TLR radiomics and OR, Combat and Limma, and different discretization methods) and the effect on superior models. The model was obtained by RF feature screening and SVM modeling, and the predicted AUC after combining clinical features was 0.811. This prediction model based on multiple data preprocessing methods can not only provide a reference for the selection of treatment options for breast cancer patients but also provide a new idea for multicenter radiomics research, which can contribute to the advancement of radiomics clinicalization.

## Supplementary Information

Below is the link to the electronic supplementary material.

Supplementary file1 (DOCX 4016 KB)

## Data Availability

The data used and analyzed during the current study are available from the corresponding author on reasonable request.
