# Supplementary material for: Development and validation of an 18F-FDG PET/CT radiomic nomogram for predicting axillary lymph-node status after neoadjuvant chemotherapy for breast cancer: a multicenter study
Source: Ann Nucl Med. 2025 Aug 17;39(12):1326–36. doi: 10.1007/s12149-025-02099-4 (PMC12602578; doi:10.1007/s12149-025-02099-4)
Supplement: Supplementary file 1 — Supplementary file1 (DOCX 4016 KB) [file 12149_2025_2099_MOESM1_ESM.docx]

**Supplementary figures and tables**

**Table A** Patient characteristics

| Characteristics | Scanner 1 | |  | Scanner 2 | |  | Scanner 3 | |  |
| --- | --- | --- | --- | --- | --- | --- | --- | --- | --- |
|  | pCR  (12) | Non-pCR  (22) | P | pCR  (29) | Non-pCR  (55) | P | pCR  (11) | Non-pCR  (18) | P |
| SUVmax | 11.75±5.76 | 10.62±3.04 | 0.472 | 10.55±6.99 | 11.44±7.41 | 0.595 | 13.88±8.52 | 8.96±4.24 | 0.056 |
| Age | 51.08±10.35 | 44.95±8.80 | 0.087 | 50.69±11.05 | 49.67±9.88 | 0.672 | 54.82±9.79 | 51.56±9.59 | 0.447 |
| Number of lymph node metastases | 3±2 | 4±3 | 0.254 | 4±3 | 5±3 | 0.144 | 4±2 | 4±3 | >0.99 |
| Lymph node metastasis size | 2.75±1.66 | 3.29±2.11 | 0.418 | 2.97±1.91 | 3.55±2.62 | 0.295 | 3.11±1.61 | 3.46±2.05 | 0.613 |
| Histologic type |  |  | 1.000 |  |  | 0.493 |  |  | 0.690 |
| IDC | 7 | 14 |  | 18 | 23 |  | 7 | 14 |  |
| Others | 5 | 8 |  | 11 | 22 |  | 4 | 4 |  |
| ER status |  |  | 0.012^＊^ |  |  | 0.001^＊^ |  |  | 0.155 |
| Positive | 2 | 15 |  | 9 | 39 |  | 3 | 11 |  |
| Negative | 10 | 7 |  | 20 | 16 |  | 9 | 8 |  |
| PR status |  |  | 0.582 |  |  | 0.01^＊^ |  |  | 0.464 |
| Positive | 2 | 7 |  | 3 | 22 |  | 1 | 5 |  |
| Negative | 10 | 15 |  | 26 | 33 |  | 10 | 13 |  |
| HER2 status |  |  | 0.062 |  |  | 0.002^＊^ |  |  | 1.000 |
| Positive | 8 | 6 |  | 19 | 15 |  | 6 | 9 |  |
| Negative | 4 | 16 |  | 10 | 40 |  | 5 | 9 |  |
| Ki-67 status |  |  | 0.789 |  |  | 0.531 |  |  | 1.000 |
| Positive | 11 | 18 |  | 26 | 45 |  | 11 | 17 |  |
| Negative | 1 | 4 |  | 3 | 10 |  | 0 | 1 |  |
| Molecular subtype |  |  | 0.034^＊^ |  |  | <0.001^＊^ |  |  | 0.115 |
| HR+ and HER2- | 1 | 11 |  | 2 | 30 |  | 1 | 7 |  |
| HER2+ | 8 | 6 |  | 19 | 15 |  | 6 | 9 |  |
| Triple-negative | 3 | 5 |  | 8 | 10 |  | 4 | 2 |  |

Notes: A t-test was used for Age and SUVmax, and a chi-square test was used for the rest.

Lymph node metastasis size is the dimension of the short diameter of the largest lymph node (unit: cm).

Results for the number of lymph node metastases are kept in whole numbers.

Abbreviations: ER, estrogen receptor; PR, progesterone receptor; HR, hormone receptor; HER2, human epidermal growth factor receptor 2; pCR, pathological complete response; IDC, Invasive Ductal Carcinoma.

^＊^ p < 0.05

**
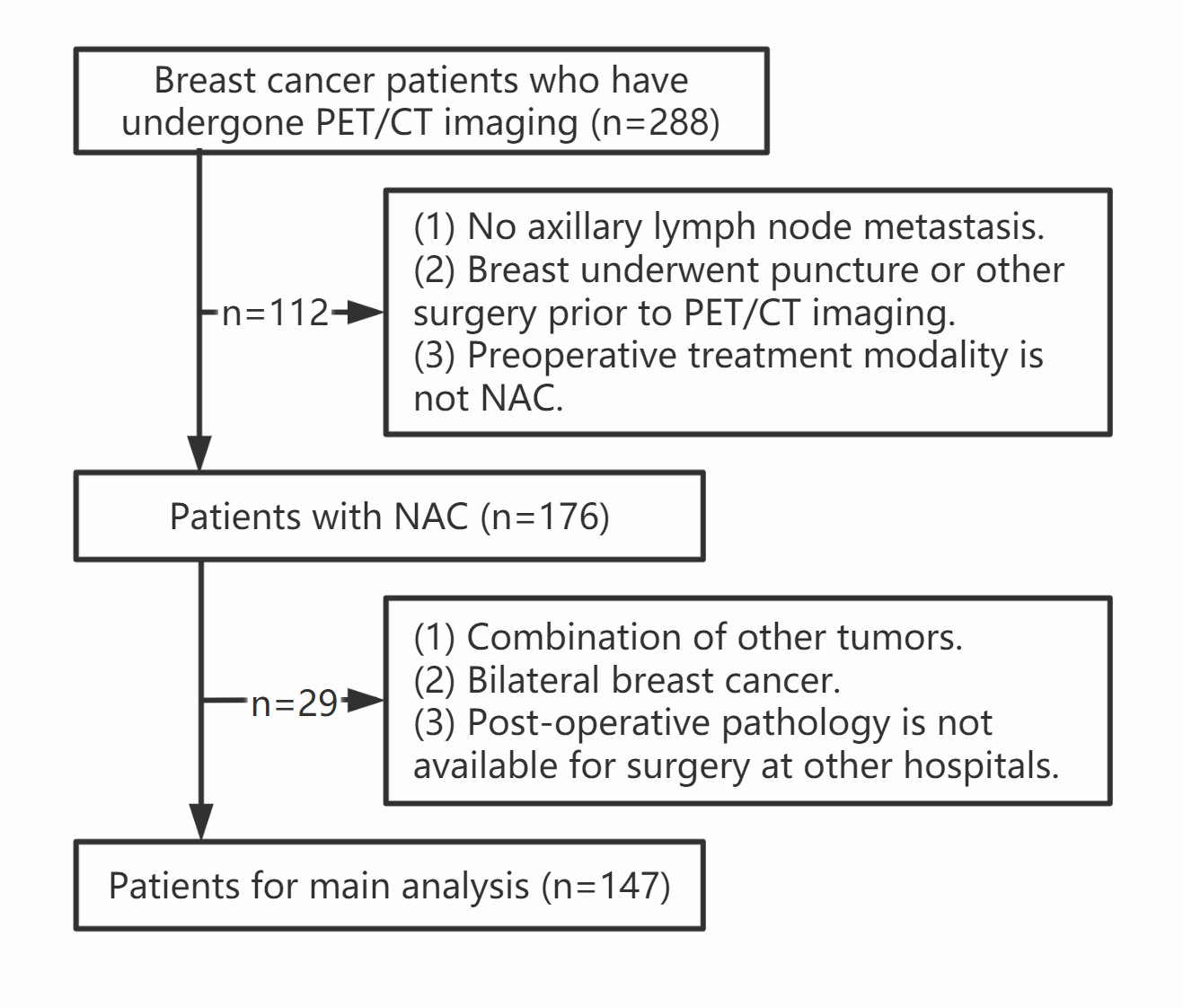
**

**Fig. A** The flowchart of patient selection.

**
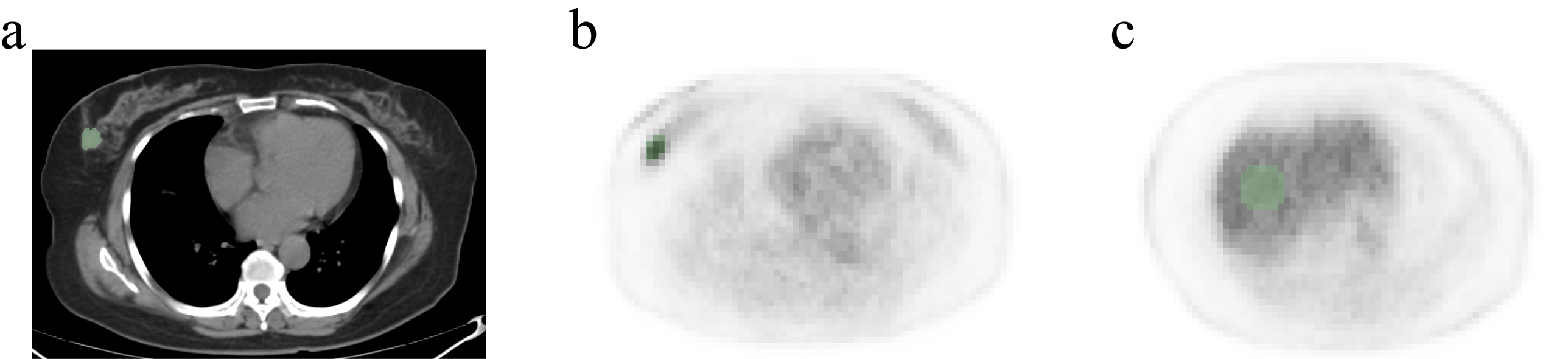
**

**Fig B** The 3D slicer was used to segment the primary lesions. **a** presents the CT image of a manually outlined lesion. **b** presents the PET image outlined by the semi-automatic thresholding method, and the threshold was 40% SUVmax. **c** presents the background of the liver outlined on a normal liver.


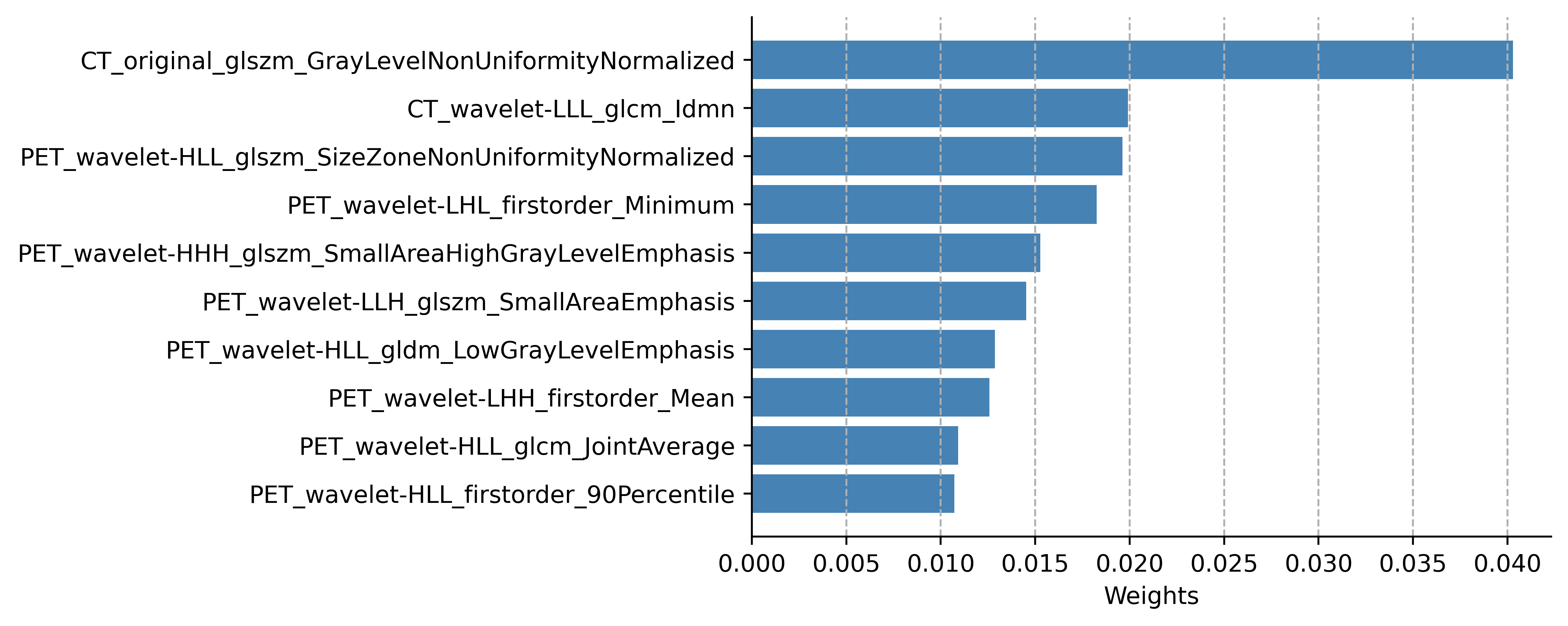
**Fig C** The names and weights of the top ten most significant features of Model 1.


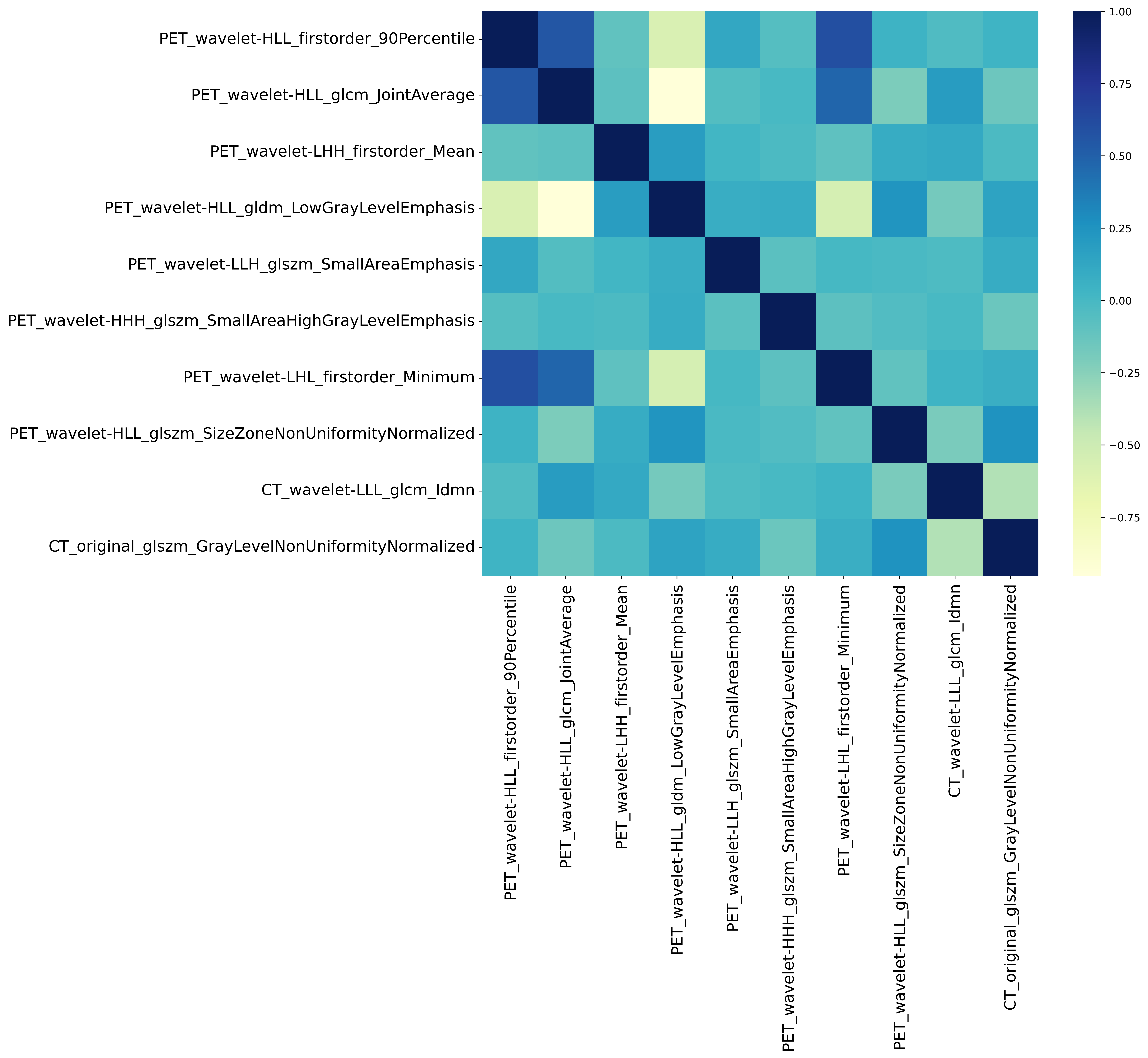


**Fig D** Heat map of correlations for the top ten most significant features of Model 1.


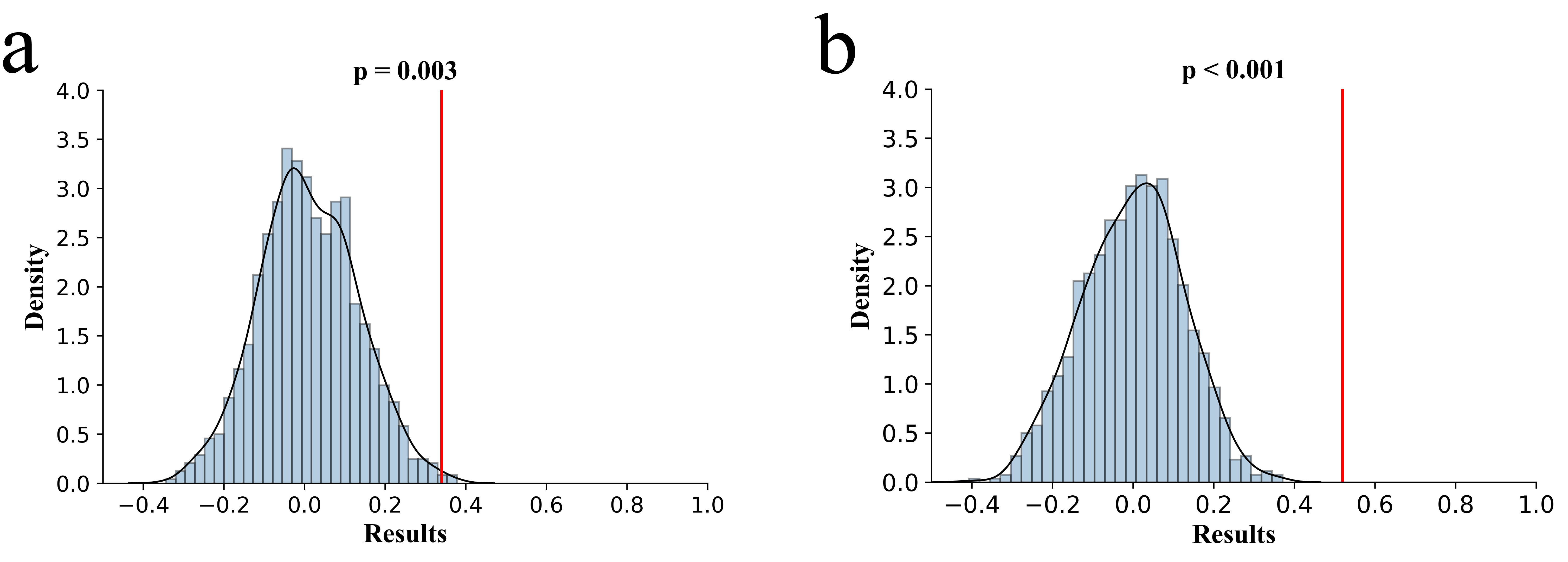


**Fig E** Permutation tests for Model 1 with the highest and lowest accuracy. **a** Permutation tests with the lowest accuracy(65.5% , p = 0.003 ＜ 0.05, Sobs = 0.340). **b** Permutation tests with the highest accuracy(96.6% , p ＜ 0.001, Sobs = 0.520).
